# Emerging Technologies and Future Directions in Interorgan Crosstalk Cardiometabolic Research

**DOI:** 10.1161/CIRCRESAHA.125.325515

**Published:** 2025-05-23

**Authors:** Hosung Bae, Christy M. Nguyen, Jorge Ruiz-Orera, Nicholas L. Mills, Michael P. Snyder, Cholsoon Jang, Svati H. Shah, Norbert Hübner, Marcus Seldin

**Affiliations:** Department of Biological Chemistry and Center of Epigenetics and Metabolism, School of Medicine, University of California Irvine School of Medicine (H.B., C.M.N., C.J., M.S.).; Cardiovascular and Metabolic Sciences, Max Delbrück Center for Molecular Medicine in the Helmholtz Association (MDC), Berlin, Germany (J.R.-O., N.H.).; BHF Centre for Cardiovascular Science (N.L.M.), The University of Edinburgh, United Kingdom.; Usher Institute (N.L.M.), The University of Edinburgh, United Kingdom.; Department of Genetics, Stanford University School of Medicine, CA (M.P.S.).; Duke Center for Precision Health (S.H.S.), Duke University School of Medicine, Durham, NC.; Duke Molecular Physiology Institute (S.H.S.), Duke University School of Medicine, Durham, NC.; German Center for Cardiovascular Research (DZHK), Partner Site Berlin, Germany (N.H.).; Charité-Universitätsmedizin, Berlin, Germany (N.H.).; Helmholtz Institute for Translational AngioCardioScience, MDC, Heidelberg University, Germany (N.H.).

**Keywords:** blood pressure, cardiovascular diseases, cell communication, endocrine system, heart diseases, hypertension

## Abstract

The heart does not work in isolation, with cardiac health and disease occurring through complex interactions between the heart with multiple organs. Furthermore, the integration of organ-specific lipid metabolism, blood pressure, insulin sensitivity, and inflammation involves a complex network of signaling pathways between many organs. Dysregulation in these communications is now recognized as a key contributor to many manifestations of cardiovascular disease. Mechanistic characterization of specific molecules mediating interorgan signaling has been pivotal in advancing our understanding of cardiovascular disease. The discovery of insulin, glucagon, and other hormones in the early 20th century illustrated the importance of communication between organs in maintaining physiological homeostasis. For example, elegant studies evaluating insulin signaling and its role in regulating glucose metabolism have shed light on its broader impact on cardiovascular health, hypertension, atherosclerosis, and other cardiovascular disease risks. Recent technological advances have revolutionized our understanding of interorgan signaling. Global approaches such as proteomics and metabolomics applications to blood have enabled the simultaneous profiling of thousands of circulating factors, revealing previously unknown signaling molecules and pathways. These large-scale studies have identified biomarkers linked to early stages of heart disease and offered new therapeutic targets. By understanding how specific cells in the heart interact with cells in other organs, such as the kidney or liver, researchers can identify key pathways that, when disrupted, lead to cardiovascular pathology. The ability to capture a more holistic view of the cardiovascular system positions interorgan signaling at the forefront of cardiovascular research. As we continue to refine our tools for mapping these complex networks, the insights gained hold the potential to not only improve early diagnosis but also to develop more targeted and effective treatments for cardiovascular disease. In this review, we discuss current approaches used to enhance our understanding of organ crosstalk with a specific emphasis on cardiac and cardiovascular physiology.

The heart does not work in isolation, with cardiac health and disease occurring through complex interactions between the heart and multiple organs, including the liver, kidneys, skeletal muscle, and adipose tissue. Interorgan signaling pathways participate in lipid metabolism, blood pressure regulation, insulin sensitivity, and inflammation, and their dysregulation is now recognized as a key driver of cardiovascular disease (CVD). For example, adipose tissue releases adipokines affecting cardiovascular function,^1,2^ and the liver regulates lipid profiles, which is critical for heart health. Both endocrine and paracrine mechanisms are important; for instance, epicardial adipose tissue, with its close proximity to the heart, exerts striking control over CVD pathobiology.^3,4^ These insights have motivated researchers to focus on systemic molecular interactions across a broad range of organs and tissues.

Mechanistic characterization of interorgan signaling molecules has been pivotal in advancing our understanding of CVD. The discovery of insulin, glucagon, and other hormones in the early 20th century illustrated the importance of organ communication in maintaining physiological homeostasis. For example, elegant studies evaluating insulin signaling have not only shed light on its role in glucose metabolism but also its broader impact on cardiovascular health, including hypertension, atherosclerosis, and other CVD risk factors.^5–7^

Recent technological advances have revolutionized our ability to study interorgan signaling. Global approaches such as proteomics and metabolomics have enabled the simultaneous profiling of thousands of circulating factors, revealing previously unknown signaling molecules and pathways linked to early stages of heart disease, offering new therapeutic targets. In addition, single-cell sequencing has facilitated high-resolution mapping of cellular composition and interorgan communication. Understanding how specific cells in the heart interact with cells in other organs, such as the kidney or liver, has revealed key pathways that, when disrupted, contribute to cardiovascular pathology.

A more integrated understanding of the cardiovascular system has positioned interorgan signaling at the forefront of cardiovascular research. As the tools for mapping these complex networks continue to improve, the resulting insights hold the potential to improve early diagnosis and development of more targeted treatments for CVD. In this review, we discuss current approaches used to enhance our understanding of organ crosstalk with a specific emphasis on cardiac and cardiovascular physiology.

## Clinical Utility of Interorgan Signaling Molecules and Potential for Discovery

The ability to interrogate the circulome using evolving technologies has provided a more comprehensive view of biological networks, facilitating mechanistic hypothesis generation and the discovery of clinically relevant biomarkers. Because blood is easily accessible, interorgan signaling molecules measurable in plasma or serum hold strong potential for clinical translation. Discovery typically begins with preclinical models or omics-based human cohort studies, followed by mechanistic validation of signaling properties and tissue sources. Interorgan signaling molecules can be translated into clinical applications in 2 key ways: as circulating biomarkers or therapeutic targets. Biomarkers may aid in diagnosis, indicating disease presence, prognosis, predicting disease outcomes, or risk assessment, estimating the likelihood of developing a disease. A biomarker may also predict therapeutic response, serve as a monitoring tool for treatment efficacy, act as a surrogate end point in clinical trials, or help distinguish disease subtypes.

Translating biomarkers into clinical use is a lengthy process, with only a few omics studies successfully advancing to clinical tests. Identifying a circulating marker in preclinical models or human cohorts is only the first step. Clinical utility requires evidence from prospective studies assessing the generalizability of the biomarker across diverse populations, the availability of therapies that modify the biomarker, and rigorous analytical validation, such as accuracy, calibration, discrimination, precision, sensitivity, and specificity. In addition, reference limits must be defined based on population characteristics, and biomarkers must demonstrate independent and incremental value over existing clinical models.^8^ Other considerations include analytical standardization, reproducibility, and both technical and biological variability, such as fluctuations due to circadian rhythms or fasting states. Practical considerations, such as cost, feasibility of clinical assays, and the need for point-of-care testing, also influence successful clinical translation.

Predating the availability of comprehensive high-throughput omics technologies, several circulating interorgan signaling metabolites and proteins have been identified as key mediators of biologic pathways integral to health and disease, with varying degrees of evidence supporting their clinical utility as biomarkers. Some examples include hormones such as insulin, thyroid hormone, and cortisol; cytokines such as interleukins and tumor necrosis factor; eicosanoids such as prostaglandins; adipokines such as adiponectin; myokines such as FGF-21 (fibroblast growth factor 21); and growth factors such as insulin-like growth factor (Table).

**Table.**
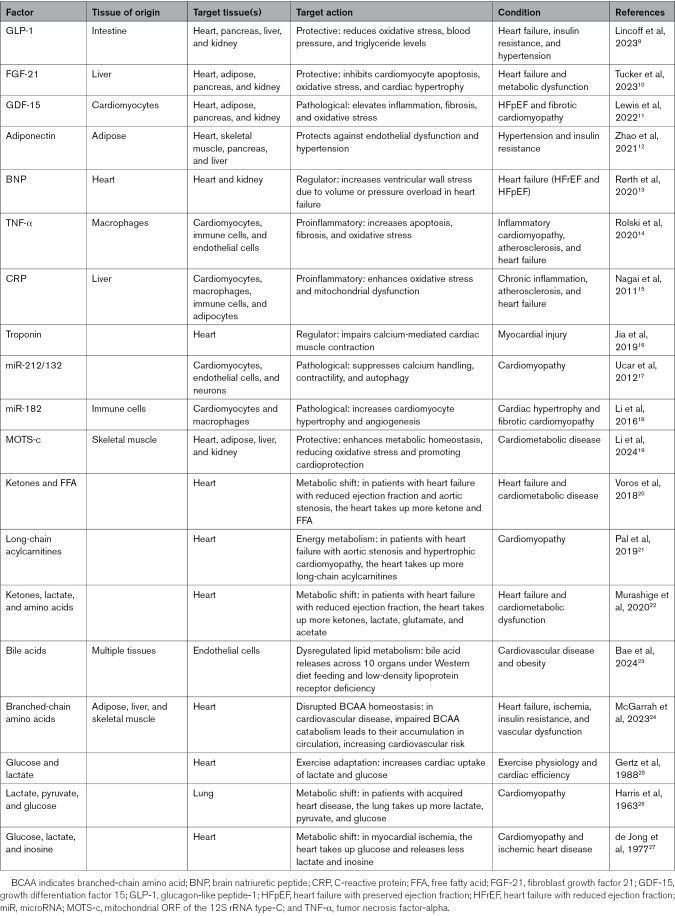
Key Focal Factors Mediating Organ Communication in Cardiometabolic Homeostasis and Disease

One of the earliest and most impactful interorgan biomarkers is glucose. First described in the early 1900s, the Cori cycle shows how lactate produced by anaerobic glycolysis in muscles circulates to the liver, where it can be recycled and converted to glucose, which is then returned to the muscle where it is metabolized to lactate.^28^ This cycle enables metabolic adaptation to energy demands. Clinically, glucose serves as a diagnostic for diabetes, a risk predictor for its development, a prognostic marker for incident cardiovascular events, a monitoring tool for insulin therapy, and a surrogate or intermediate end point in diabetes studies. A more recent example of an interorgan signaling molecule that has widespread clinical utility is BNP (brain natriuretic peptide) and related NT-proBNP (N-terminal pro-B-type natriuretic peptide). BNP and NT-proBNP are secreted by cardiac ventricles and have systemic effects, including inducing natriuresis, diuresis, and renin-angiotensin-aldosterone system inhibition in the kidneys; producing vasodilation in blood vessels; reducing aldosterone secretion in adrenal glands; inhibiting sympathetic tone in the brain; relieving congestion in the liver; and decreasing pulmonary pressures in the lungs.^29^ Clinically, BNP and NT-proBNP are widely used to diagnose heart failure, predict incident cardiovascular events, and monitor therapeutic response to diuretic therapy.

Recent large-scale discovery efforts such as proteomics have identified interorgan signaling molecules with clinical utility as both biomarkers and therapeutic targets. Examples include FGF-21 and GDF-15 (growth differentiation factor 15). FGF-21, a hepatokine, was first identified as a novel metabolic regulator through a phenotypic screen testing novel secreted proteins for their ability to enhance glucose uptake in mouse adipocytes.^30^ Subsequent studies showed that FGF-21 exerts metabolic protective effects through multiple target tissues, including the brain, liver, adipose tissue, pancreas, cardiac tissue, muscle, intestines, and vasculature.^31^ Findings from large-scale meta-analysis studies in humans found that serum FGF-21 is linked to type 2 diabetes, metabolic dysfunction–associated steatotic liver disease, atherosclerosis,^32^ and heart failure.^33,34^ However, its prognostic value for incident cardiovascular events^35^ remains unclear, as studies have produced conflicting results independent of traditional risk factors. While some CVD studies have evaluated the discriminative and prognostic utility of FGF-21 beyond obesity and metabolic dysfunction–associated steatotic liver disease, further large-scale studies are necessary to validate the clinical utility and diagnostic or prognostic thresholds.

Despite biological significance, some interorgan signaling molecules that initially showed promise for clinical use ultimately failed to translate into viable biomarkers. TNF-α (tumor necrosis factor-alpha), an inflammatory cytokine produced by immune cells and adipose tissue, has effects on endothelial cells, hepatocytes, cardiomyocytes, and neurons, triggering inflammatory responses, promoting insulin resistance and vascular dysfunction. Early studies linked circulating TNF-α levels to heart failure^36^ and suggested its potential as a biomarker based on changes observed with heart failure therapies.^37^ However, TNF-α ultimately was not adopted for clinical use due to its nonspecificity for CVD, complex role in cardiac function, high biological variability, and discrepancies between circulating and tissue expression levels. Similarly, adipokines, which mediate adipose tissue signaling with the liver and cardiovascular system, have faced challenges in clinical translation. For example, adiponectin exhibits high variability in measurements and overlaps with metabolic syndrome biomarkers, limiting its utility as a cardiometabolic disease biomarker.^2^ Given the well-established roles of validated CVD biomarkers such as troponin,^38^ NT-proBNP, and CRP (C-reactive protein),^39^ the threshold remains high for introducing new biomarkers into clinical application. Nonetheless, advancements in high-throughput discovery platforms continue to accelerate the identification of interorgan signaling molecules with potential clinical utility.

Beyond biomarker discovery, these technological advances also yield new therapeutic targets, either through the development of novel pharmaceutical agents or repurposing of existing drugs. These targets may involve circulating interorgan signaling molecules that drive disease when dysregulated or pathological interorgan communication pathways that can be therapeutically modulated. For example, targeting cytokine signaling has shown effective in autoimmune diseases, while targeting metabolic pathway modulation has become a key approach in diabetes therapeutics. For instance, FGF-21 has not only shown promise as a clinical biomarker but also as a therapeutic target. Over the past decade, several FGF-21 derivatives and FGF-21 receptor agonists have been developed and tested as therapeutic agents for various metabolic disorders including type 2 diabetes, obesity, and metabolic dysfunction–associated steatotic liver disease.^40^ Perhaps, the most transformative therapeutics to date in cardiometabolic diseases are those targeting interorgan signaling molecules. GLP-1 RAs (glucagon-like peptide-1 receptor agonists), originally developed as antiglycemic agents, mimic GLP-1 (glucagon-like peptide-1), a hormone secreted by the intestine that has interorgan effects on the heart, liver, kidney, and adipose tissue, regulating glucose metabolism, appetite, gastric emptying, and cardiovascular function. GLP-1 RAs have shown cardiometabolic benefits in both patients with diabetes and without diabetes and have shown efficacy across a wide variety of CVDs.^41^

It is important to note that the ability of a circulating biomarker to show clinical utility as a diagnostic, prognostic, or disease-related biomarker does not directly translate into serving as an efficacious therapeutic target. Biomarkers may fail as therapeutic targets due to several reasons, including being a by-product of the disease or related risk factor, and, thus, not causal for the disease itself; pleiotropic effects leading to an imbalance of adverse to beneficial effects; off-target drug toxicities; or nonspecific effects with diverse downstream effects. As a case in point, therapies targeting the previously mentioned TNF-α showed both pathological and protective effects in CVD. ^42^The potential reason for this is thought to be related to the complex role of TNF-α in the cardiovascular system, with mixed effects depending on different TNF receptor interactions. For instance, excessive inhibition of TNF-α may disrupt TNFR2 signaling, which has cardioprotective functions.^43^

## Technological Advances to Deconvolute Organ Signaling

Recent technological advances have expanded our ability to map the circulating circulome, revealing the intricate network of factors that signal and exchange between organs. For example, mass spectrometry (MS)–based quantification of organ exchange expands our knowledge of organ communication beyond a static snapshot to active functions. These innovations hold significant potential for unraveling complex metabolic pathways and understanding their influence on health and disease. Tracing metabolites across organs not only reveals systemic responses to dietary interventions but also deepens our comprehension of metabolism in conditions such as CVD (Table).^44^ For instance, CVD often stems from arterial blockages caused by metabolites such as oxidized cholesterol, which are metabolized by organs such as the liver, intestines, adipose tissue, and skeletal muscles.^45^ This highlights the vital interplay among these organs and emphasizes the importance of exploring metabolite exchange between the heart and other tissues.^46^ Deciphering these dynamics further can illuminate how metabolic dysregulation and disrupted interorgan crosstalk drive CVD, paving the ways for therapeutic innovations.

**Figure 1.**
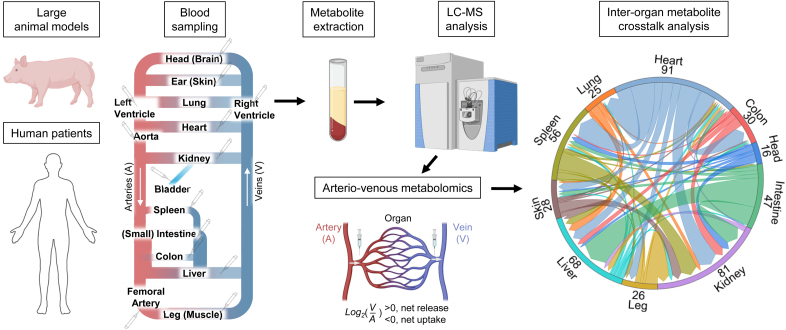
**Arteriovenous metabolomics reveals interorgan flux.** The workflow of arteriovenous metabolomics involves using large animal models, such as pigs, or human patients. Venous and arterial blood samples collected from sites representing different organs are extracted for metabolite measurement by liquid chromatography-mass spectrometry (LC-MS). By comparing metabolite levels in arterial (A) and venous (V) blood, the net production or consumption of metabolites by each organ can be assessed. A higher venous concentration (V>A) indicates net metabolite release by the organ, whereas a lower venous concentration (V<A) signifies net metabolite uptake. Metabolite exchange between organs can be visualized on the right, with numbers highlighting significant exchanges for each organ.

To effectively prevent the accumulation of metabolites that contribute to CVD risk, it is crucial to identify the organs primarily responsible for their production and consumption and to determine their quantitative contributions. Traditional omics approaches, such as transcriptomics, proteomics, and metabolomics, are limited in this regard as they provide static snapshots of metabolite levels rather than dynamic fluxes that capture rates of metabolite release and uptake by organs.^47^ Arteriovenous measurements have an advantage in their ability to infer organ-specific metabolic flux by elucidating the metabolite net uptake or release of each organ (Figure 1). Enhanced by recent revolutionary technological advancements in MS, arteriovenous metabolomics now enables simultaneous measurement of hundreds to thousands of metabolites, offering comprehensive insights into cross-organ metabolic disruptions underlying CVD processes. For example, studies by Voros et al^20^ and Pal et al^21^ have revealed distinct metabolic patterns in conditions such as aortic stenosis and hypertrophic cardiomyopathy, including increased free fatty acid and long-chain acylcarnitine utilization by the heart. Using arteriovenous metabolomics in human patients, measurements of over 270 metabolite fluxes have revealed the preference of the heart for fatty acid utilization in healthy individuals. However, during heart failure, there is a shift toward increased consumption of ketones and lactate.^22^ By leveraging large animal models that recapitulate human CVD pathophysiology, researchers have further applied arteriovenous metabolomics to simultaneously measure metabolic fluxes across multiple organs. For instance, using pig models, researchers have identified disrupted metabolite distributions and hormonal signaling across 10 organs under Western diet feeding and low-density lipoprotein receptor deficiency.^23^ These discoveries underscore the complex interorgan metabolic interactions that drive CVD pathophysiology.

Beyond interorgan fluxes, advancing cardiovascular health also requires deciphering interorgan metabolism and the roles of circulating factors in specific cells and tissue regions, and their involvement in signaling pathways and homeostasis. In this context, arteriovenous metabolomics provides only net fluxes (production minus consumption) across a target organ, without the ability to distinguish intraorgan fluxes (eg, gluconeogenesis in the kidney cortex versus glycolysis in the medulla) or region-specific activities (eg, femoral vein blood from skeletal muscle, adipose tissue, bone, and skin). Recent technological innovations address these gaps with tools such as stable isotope tracing, combined with arteriovenous measurements and mathematical modeling, to quantify intraorgan gross fluxes.^47^ Single-cell metabolomics offers granular insights, and advances in cell isolation methods enhance its utility.^48^ Imaging MS, such as matrix-assisted laser desorption/ionization imaging MS, further maps metabolite distributions spatially.^49^ Integrating these approaches enables a deeper understanding of metabolic fluxes at the cellular and regional levels, offering critical insights into CVD mechanisms and potential therapeutic targets.

Building on the growing recognition of the importance of the circulating milieu, the exploration of unannotated short open reading frames (sORFs) has gained momentum.^50^ Only recently have we been able to map sORFs outside of cells, which are typically <100 amino acids, encoded within untranslated regions of protein-coding mRNAs and long noncoding RNAs. A pivotal advancement in this field has been the development of ribosome profiling, a technique that precisely identifies actively translated regions of the transcriptome.^51^ By sequencing ribosome-protected RNA fragments, this method provides a high-resolution snapshot of translation, revealing the exact positions of ribosomes on RNA transcripts. Recent studies using this technology have identified hundreds of translated sORFs across human organs, including the heart, kidney, brain, and liver.^52–54^ Many of these human sORFs display organ- and species-specific translation^54–56^ are dysregulated in conditions such as heart failure^5^ and play roles in metabolic pathways such as oxidative phosphorylation and mitochondrial function,^52,54,57–60^ emphasizing their potential relevance to CVD.

**Figure 2.**
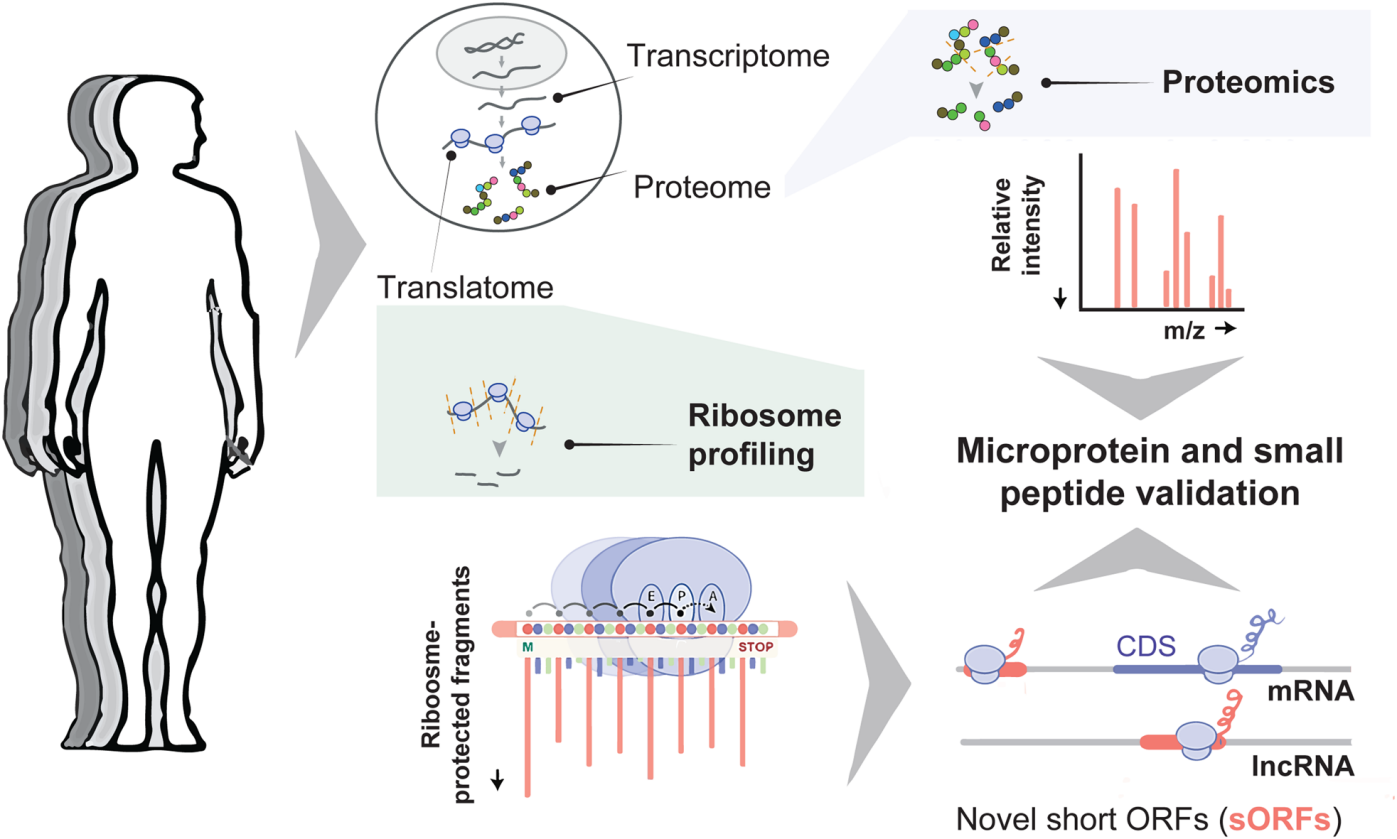
**Detection of microproteins and small peptides.** Simplified workflow for detecting microproteins and small peptides from human samples. The focus is on the translatome, which reflects all actively translated RNA sequences, studied through ribosome profiling. This technique captures ribosome-protected RNA fragments, enabling the identification of actively translated regions, including novel short open reading frames (sORFs). These sORFs, found in untranslated regions of mRNAs and long noncoding RNAs (lncRNAs), can be validated as encoding microproteins and small peptides through proteomic analyses. This combined approach of ribosome profiling and proteomics facilitates high-throughput detection of new potential circulating microproteins and small peptides.

While ribosome profiling is a powerful tool for detecting active sORF translation, it does not directly identify their encoded protein products. Complementary proteomics approaches, such as liquid chromatography-tandem MS, selected reaction monitoring, and parallel reaction monitoring, provide direct evidence of protein synthesis from whole-cell or selected sORFs.^61,62^ The integration of ribosome profiling with MS-based methodologies has proven to be an effective strategy for identifying stable microproteins by helping to define the search translated space,^63–66^ including potential elements that circulate in the bloodstream (Figure 2). For instance, MS analysis of purified extracellular vesicles has detected microproteins within these vesicles, suggesting novel mechanisms for intercellular communication.^67^ Furthermore, MS-based analysis under various physiological and dietary conditions in mice has revealed 33 microproteins present in the secretome of adipose tissue, these microproteins often being coexpressed with key metabolic genes.^68^ In addition, MS-based methods can be used to predict the functionality of products encoded by sORFs. For instance, the protein interaction screen on the peptide matrix provides a scalable solution for characterizing protein-protein interactions, facilitating high-throughput mapping of protein interactomes. This method has demonstrated functional roles for small peptides as short as 5 amino acids, highlighting their capacity to engage in significant biological processes by interacting with larger protein complexes and modulating cellular functions such as mRNA splicing, endocytosis, and translation regulation.^63^

As our understanding of these overlooked elements of the genome continues to expand, their involvement in key signaling pathways and tissue homeostasis holds promise for novel therapeutic strategies targeting multiorgan disorders. One promising example is MOTS-c (mitochondrial ORF of the 12S rRNA type-C), a 16-amino amino acid microprotein encoded by mitochondrial DNA that plays a critical role in regulating insulin sensitivity and glucose metabolism.^69^ Circulating in the bloodstream as a mitochondrial-derived peptide hormone, MOTS-c primarily influences skeletal muscle and heart tissues by activating AMPK (AMP-activated protein kinase) and maintaining metabolic balance. In mouse models, MOTS-c treatment enhances insulin sensitivity under high-fat diet conditions, reduces obesity risk, and prevents lipid accumulation in the liver. The discovery and characterization of additional sORF-encoded microproteins and small peptides, thus, have the potential to unveil novel regulatory mechanisms with implications for cardiovascular health.

Despite promising bench-side discoveries through advanced technologies, not all biomarkers successfully translate into clinical practice. For example, branched-chain amino acids, although closely linked to cardiometabolic risk,^24^ have not been adopted clinically due to limited prospective validation, assay complexity, high costs, and unclear therapeutic relevance. Conversely, CRP has successfully become a clinical biomarker because of robust validation in large-scale epidemiological trials (eg, JUPITER [Justification for the Use of Statin in Prevention: An Intervention Trial Evaluating Rosuvastatin]^70^), widely accessible assays (eg, ELISA [Enzyme-Linked Immunosorbent Assay]), and well-defined therapeutic implications. Thus, translating novel technologies into clinical use requires strong validation, assay accessibility, affordability, and clear therapeutic pathways.

## Leveraging Differences Between Individuals to Define Organ Crosstalk

Over the past 2 decades, an explosion of new resources encompassing multiomic data has been generated, enabling the development of analysis-based tools focused on studying how organs communicate. These bioinformatic tools provide a powerful framework for understanding interorgan crosstalk by leveraging genetic and cardiometabolic variation across individuals and environmental settings. Initial approaches arose from the concept that variation in plasma omics data presents strong predictive value for disease progression. This concept was further supported by the paralleled accessibility of human blood and continuous cost reductions with assaying genomic variation, enabling a rapid expansion of associations between the genome and circulating proteins (protein quantitative trait loci or metabolites). Several intriguing approaches have been used to refine these associations in the context of CVD, including Mendelian randomization,^71^ colocalization of plasma associations with tissue-specific expression QTLs,^72^ and personalized-risk scores based on circulating associations.^73^ The vast number of resulting associations and potential causal mechanisms of human CVD gave rise to questions about the mechanism of action. When an association between a circulating factor in the blood occurs is this a direct or indirect association? Furthermore, is the association due to a dysregulated ability to produce or resistance to actions?

**Figure 3.**
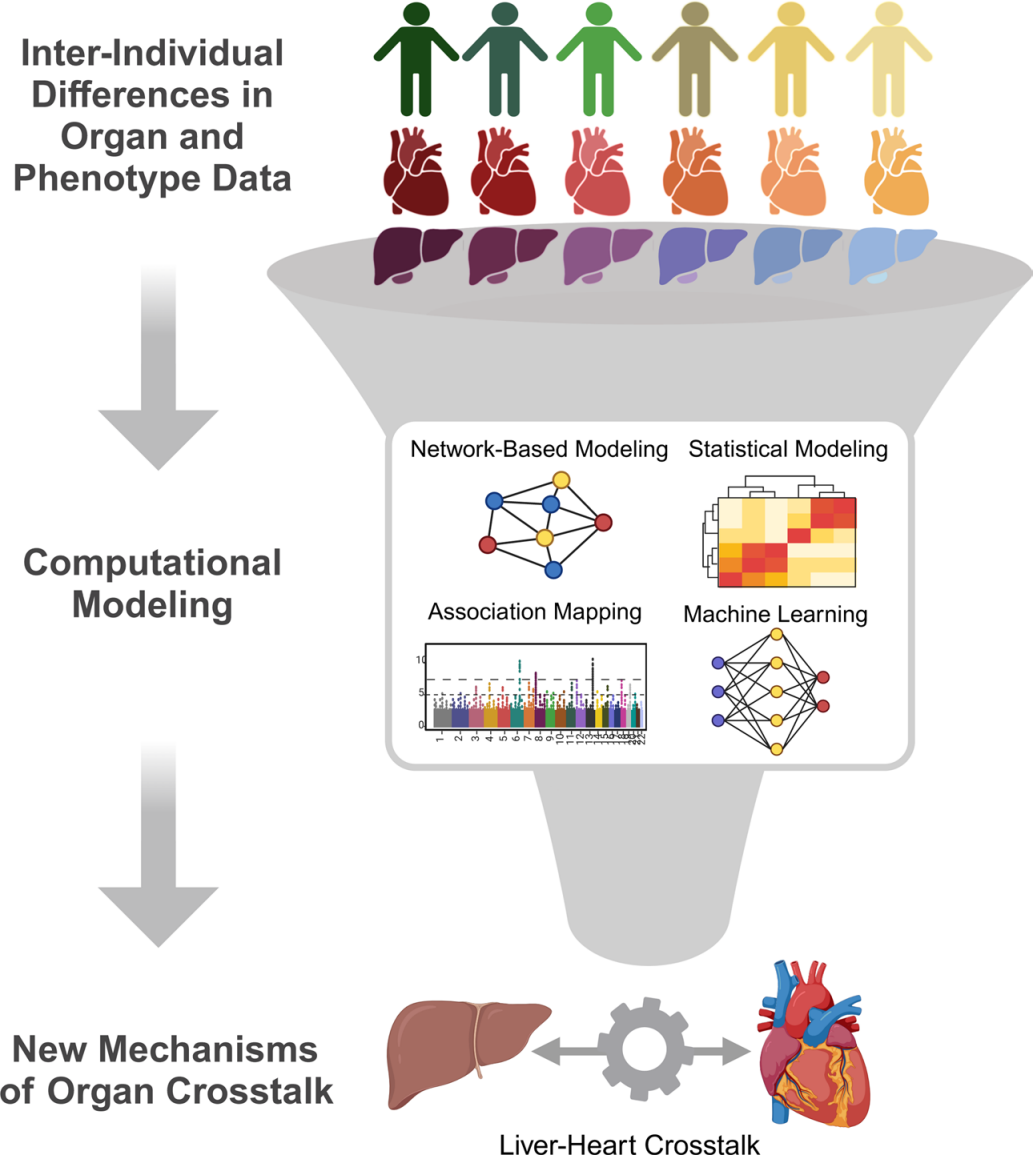
**Population-based approaches for discovery of organ crosstalk mechanisms.** In a population where heterogeneity of organ-level omic data (eg, RNA-seq or proteomics) is observed (**top**), these differences can be analyzed using network-based or statistical modeling approaches (**middle**) to uncover new modes of tissue communication (**bottom**).

Systematic analyses explicitly focused on mechanisms of organ crosstalk are required to address these questions. An initial key approach to narrow relevant mechanisms of organ communication in CVD applied a network-based aggregation approach, weighted gene coexpression network analysis, to refine a multitissue coexpression to discrete models of communication.^74^ The intuition for this approach was simple that individual covariation of gene expression networks in a population was sufficient to partition tissue communication circuits into discrete functional modules. Modules of shared tissue coordination could be further refined by integrating other data such as traits (eg, circulating LDL-C [low-density lipoprotein (LDL) cholesterol]). This approach was refined by constructing Bayesian network models and asking which genes showed the strongest centrality to shared tissue models, thus prioritizing potential mediators of communication.^75–77^ More recent elegant expansions of this intuition have provided new frameworks to study organ signaling, such as analyses of conservation of associations across human and mouse diversity, and have unveiled new mechanisms of CVD.^78,79^ Specifically, von Scheidt et al^79^ compiled a database of all genes in mice, where genetic ablation impacts atherosclerosis development.^79^ This list of genes was integrated with mouse and human diversity of gene expression and phenotypic responses using the Mergeomics^80^ framework to pinpoint the core molecular underpinnings of atherosclerosis. The intuition that mechanisms of organ crosstalk could be easily identified through analyses of covariation between tissues in a population has led to the development of new methods to leverage variation for the discovery of tissue communication mechanisms. For example, our groups showed that systematic surveys of correlation structure from multitissue sequencing data were sufficient to elucidate new proteins involved in organ communication.^81–83^ The intuition behind this approach is simple in that molecules mediating signaling between organs, as well as mechanisms of action, show individual differences and, therefore, appear as strongly significant outcomes when surveying global correlation structure (Figure 3). For example, Cao et al^83^ searched for mechanisms of liver-heart communication leveraging global correlation structure and defined a new role for factor XI as a liver-derived mediator of heart function in models of heart failure with preserved ejection fraction.^83^ This concept has been used similarly for single-cell sequencing data sets (compared and reviewed in the study by Wilk et al^84^ and Dimitrov et al^85^), whereby methods enable users to look at changes in known ligand-receptor pairs in the context of relevant comparisons such as differential expression between conditions. The recent development of multiorgan single-cell data sets has enabled the expansion of this approach across tissues.^86^ Additional methods have since been developed to search for mechanisms of organ crosstalk using models that focus on centrality or literature-based mining.^87–89^ Context-dependent interactions between organs using population-based approaches present an exciting new area to focus these tools. In this issue, Strocchi et al^90^ leverage mouse and human diversity alongside sequencing data from heart failure with preserved ejection fraction to identify cross-organ pathways and signals conserved across species.^90^ Recent models have adopted this framework to target organ coordination mechanisms, which defer depending on other contexts such as aging^91^ or sex differences.^92^ Undoubtedly, continuous refinement of population-based models focused on organ crosstalk will help to understand how tissue signaling is altered in disease states and prioritize new therapeutic candidates.

Beyond the identification of specific signaling circuits and contexts influencing organ crosstalk, circulating factors, such as proteins or metabolites, present significant potential as personalized predictors of disease. Specifically, several studies have highlighted the utility of these biomarkers in identifying individual-specific disease risks, offering a window into the systemic effects of localized dysfunction. In a key study that sets a strong foundation for plasma profiling to personalize our understanding of the disease, Chen et al^93^ performed deep multiomic profiling in patients to develop personalized signatures for pan-disease predictions.^93^ Since then, many studies have validated and refined this approach to leverage the vast amount of high-throughput data in plasma to individualize disease prediction.^94,95^ Examples include stratifying plasma protein levels by severity of heart failure outcomes,^96^ as well as integrating mouse and human diversity panels to understand mechanisms spanning individual differences.^97^ In a general sense, the potential of detailed multiomic and analytical applications in a single individual across time and diverse exposures presents significant appeal in understanding how changes in these markers relate to disease progression. These types of analyses also have the appeal of escaping complexities associated with the highly variable nature of data derived from interindividual differences, where contextualizing the relative genetic, environmental, and interactions influencing variance remains incredibly challenging.

## Future Directions in Interorgan Signaling Research

Advancements in high-throughput technologies, large-scale cohort studies, and computational tools have allowed unprecedented insights into interorgan signaling. However, only a small fraction of the interactome mediating complex cardiometabolic homeostasis has been elucidated, requiring continued revolutions in system-level approaches to translate multiomics research into clinical applications. Efforts such as the UK Biobank^98^ and All of Us^99^ have pioneered new templates to leverage interindividual differences to the heterogeneity of disease, but a considerable limitation remains in the ability to define direct genotype-to-phenotype relationships. Notably, these resources present a definitive lack of organ-specific functional and molecular measures. Although plasma proteomics has advanced biomarker discovery and several computational approaches have emerged to classify organ-specific signatures of biomarkers,^91,100^ they still lack the critical resolution necessary to define precise organ contributions to disease pathology. Future efforts should focus on developing resources that provide measures from the same organs or cellular-resolved pan-organ data similar to the Genotype-Tissue Expression resource, which has revolutionized our understanding of organ-specific contributions to disease, empowering other researchers in using these data.^101^

Current resources for studying interorgan signaling rely on genetic diversity or individual heterogeneity to uncover underlying mechanisms. Developing multiorgan resources that incorporate these variables will be essential for advancing analyses of organ crosstalk. While this approach presents strong validity, it can overlook temporal dynamics, environmental variables, or complex interactions such as gene-by-diet effects, all of which are known to shape how organs communicate. For example, a recent comparative analysis of diverse inbred strains and dietary responses demonstrated the importance of considering gene-by-diet interactions in insulin action on cardiometabolic organs.^102^ In this light, genetic reference panels, such as those from rodent models or human cell lines, enable the same genetic background to be studied under varying environmental or temporal exposures, enabling quantification of shifts in interactions in the context of cell or organ signaling. Recent advancements in the availability of single-cell sequencing have also led to the development of computational tools, which investigate ligand-receptor interactions in single-cell databases, discussed above. While these frameworks could easily be repurposed to explore interorgan signaling, heavy reliance on the use of annotated ligand-receptor pairs remains, limiting the quality, generalizability, and context dependence of predictions. Many existing databases rely on pathway references, yeast-2-hybrid assays, and cross-linked protein-protein interaction data from cells.^103–105^ However, newer approaches are being developed to enable direct evaluation of protein and chemical interactions across cells or native tissue environments,^106–108^ thus opening up new avenues for exploration of discrete signaling mechanisms in relevant physiological contexts.

Refinement of tools and assays used to measure molecules mediating communication will further provide key insight as to which factors or conditions are more relevant and selective for cardiometabolic disease. The expansion of MS methods to trace and quantify metabolites and proteins mentioned above presents thousands of new windows to explore mechanisms of organ crosstalk. In addition to these methods, sequence-based refinement elucidating how the genome is regulated will lead to the characterization of new signaling molecules. For example, usage of long-read sequencing, computational models, and new experimental model systems have identified novel isoforms or even transcriptional products, which could serve as new signaling molecules.^109–112^ For instance, growing areas of interest include extracellular vesicles,^113^ micropeptides,^114^ microRNAs,^115^ brown adipose tissue-derived adipokines (batokines),^116^ and gut microbiome-derived metabolites such as short-chain fatty acids.^117^ Extracellular vesicles have also emerged as carriers of cargo, which can communicate between cells and are altered in CVD.^118,119^ While comprehensive characterization of extracellular vesicle signaling remains limited due to technical constraints,^120–122^ studies on these molecules further present exciting opportunities.

Despite significant advances, translating findings on interorgan crosstalk in CVD into clinical practice remains challenging due to hurdles related to validation, technological feasibility, and economic constraints. Many promising biomarkers identified through preclinical studies fail to consistently demonstrate clinical relevance in larger, more diverse patient populations, limiting their clinical translation.^123^ Advanced technologies introduced in this review, including MS, single-cell sequencing, ribosome profiling, and various multiomic platforms, also encounter difficulties in standardization, complexity, and reproducibility. Economic factors further restrict translation, as the high costs and specialized nature of these assays make routine clinical adoption difficult. Overcoming these barriers involves developing standardized assays and scalable, cost-effective technologies that can be readily adopted by clinical laboratories. In addition, extensive clinical validation studies are essential,^70,124,125^ along with clearly demonstrating the therapeutic value and practical implications of these preclinical findings to inform and enhance clinical decision-making.

Computational modeling, AI, machine learning, and network-based approaches have opened up new frontiers in precision medicine and transformative health care. In particular, tools that simultaneously model genetics and multitissue data will serve to define relevant pathways for CVD. An elegant example is Mergeomics^80^ that enables researchers to query genetic multitissue data, pharmacological interactions, and key drivers. Similar powerful and accessible tools will assist in the deconvolution of organ communication relevant to CVD and other conditions. Machine learning will continue to play a key role in these approaches; however, the ability to test the replication, validity, and utility of machine learning models must be a key consideration in these developments. For example, AlphaFold, developed by Google, uses AI to predict the 3-dimensional structures of proteins with a high degree of accuracy,^126^ facilitating small molecule development. A growing arsenal of druggability databases enables researchers to assess the suitability of a new protein as a target for drug development. With emerging technologies and expanding data sets to survey molecules and pinpoint key players in CVD pathophysiology across genetic and environmental differences, the future of interorgan signaling research holds immense potential for translating discoveries into clinical impact. These efforts combined with collaborative team science, an iterative cycle of bench-to-bedside, and a culture of data sharing will advance the evaluation of tissue sources, mechanisms, and clinical utility to benefit public health.

## ARTICLE INFORMATION

### Acknowledgements

Illustrations were created with BioRender.com.

### Sources of Funding

M.S. was supported by NIH grant DP1DK130640 and Novo Nordisk Foundation. N.H. was supported by the Chan Zuckerberg Initiative; the Leducq Foundation (16CVD03); the British Heart Foundation and Deutsches Zentrum für Herz-Kreislauf-Forschung (BHF/DZHK: SP/19/1/34461); the DFG SFB-1470 (project B03); an ERC-advanced grant under the European Union Horizon 2020 Research and Innovation Program (AdG788970). N.L.M. is supported by a Chair Award, Programme Grant, and Research Excellence Award (CH/F/21/90010, RG/20/10/34966, RE/24/130012) from the British Heart Foundation.

### Disclosures

None.
